# Breast Reconstruction Does Not Affect the Survival of Patients with Breast Cancer Located in the Central and Nipple Portion: A Surveillance, Epidemiology, and End Results Database Analysis

**DOI:** 10.3389/fsurg.2022.855999

**Published:** 2022-05-17

**Authors:** Mingchen Xiong, Zeming Liu, Wenchang Lv, Chongru Zhao, Yichen Wang, Yufang Tan, Qi Zhang, Yiping Wu, Hong Zeng

**Affiliations:** Department of Plastic and Cosmetic Surgery, Tongji Hospital, Tongji Medical College, Huazhong University of Science and Technology, Wuhan, China

**Keywords:** breast reconstruction, mastectomy, Surveillance Epidemiology and End Results database, breast cancer, prognosis

## Abstract

**Background:**

Tumors in the central and nipple portion (TCNP) are associated with poor prognosis and aggressive clinicopathological characteristics. The availability and safety of postmastectomy reconstruction in breast cancer patients with TCNP have still not been deeply explored. It is necessary to investigate whether reconstruction is appropriate for TCNP compared with non-reconstruction therapy in terms of survival outcomes.

**Methods:**

Using the Surveillance, Epidemiology, and End Results (SEER) database, we enrolled TCNP patients diagnosed between the years 2010 and 2016. The propensity score matching (PSM) technique was applied to construct a matched sample consisting of pairs of non-reconstruction and reconstruction groups. Survival analysis was performed with the Kaplan–Meier method. Univariate and multivariate Cox proportional hazard models were applied to estimate the factors associated with breast cancer-specific survival (BCSS) and overall survival (OS).

**Results:**

In the overall cohort, a total of 6,002 patients were enrolled. The patients in the reconstruction group showed significantly better BCSS (log-rank, *p* < 0.01) and OS (log-rank, *p* < 0.01) than those in the non-reconstruction group (832 patients) after PSM. However, the multivariate Cox regression model revealed that breast reconstruction was not associated with worse BCSS and OS of TCNP patients.

**Conclusion:**

Our study provided a new perspective showing that breast reconstruction did not affect the survival and disease prognosis in the cohort of TCNP patients from SEER databases, compared with non-reconstruction. This finding provides further survival evidence supporting the practice of postmastectomy reconstruction for suitable TCNP patients, especially those with a strong willingness for breast reconstruction.

## Introduction

Breast cancer (BC) is the most commonly diagnosed cancer and the main cause of cancer death for women worldwide, overtaking lung cancer as the leading cause of global cancer incidence in 2020 (1). Owing to different clinical presentation and molecular features, the occurrence and prognosis of BC are related to patients and tumor characteristics such as patient gender, age, family history, hormone receptor status, tumor size, lymph node involvement, and histologic grade (2–4). Of note, the primary tumor location might be an important feature affecting BC prognosis (5). According to a survey, BC originating from the central portion had the highest mortality and a higher risk of recurrence, and tumors from the central and nipple portion were related to the presence of positive lymph nodes (6). Besides, the tumors in the central and nipple portion (TCNP) of BC had adverse effects on breast cancer-specific survival (BCSS) and overall survival (OS) rate, compared with the tumor in the breast peripheral quadrant (7).

Generally based on Tumor–Node–Metastasis (TNM) staging and molecular markers, BC prognosis and treatment options are breast-conserving surgery, radiation therapy, adjuvant systemic therapy, chemotherapy, endocrine therapy, and induction chemotherapy, followed by mastectomy (8, 9). However, mastectomy, low or centrally located cancer, large-sized breasts with breast ptosis, and traditional BC treatment can all lead to unacceptable cosmetic outcomes (10). Postmastectomy reconstruction is regarded as a coping strategy in the therapeutic course following BC and mastectomy, which helps women to overcome body image disturbance (11). Due to poor TCNP prognosis and more aggressive clinicopathological characteristics, the availability and safety of postmastectomy reconstruction in patients with TCNP have still not been deeply explored. In addition, studies concerning a comparison of prognosis and survival between non-reconstruction and reconstruction therapy for TCNP patients have rarely been discussed.

Herein, we conducted this study using the Surveillance, Epidemiology, and End Results (SEER) database to describe postmastectomy reconstruction and discuss the underlying prognostic indicators and outcomes, intending to shed light on the survival and effects of postmastectomy reconstruction in BC patients with TCNP.

## Materials and Methods

### Patients

The clinicopathological features and survival data from 2010 to 2016 were collected using the SEER database. This database collected data on patient demographics, tumor characteristics, the first course of treatment, and follow-up for vital status (12). The case listing in this study was generated by using SEER∗Stat software (version 8.3.9.2). Considering the available HER2 information and data heterogeneity decrease, we collected the SEER patients diagnosed between 2010 and 2016. To investigate the clinicopathological characteristics of postmastectomy non-reconstruction and reconstruction of patients with TCNP, the following information was obtained: reconstruction surgery, region, insurance, age, race, marital status at diagnosis, year of diagnosis, laterality, grade, AJCC stage, tumor size, LN status, ER status, PR status, HER2 status, radiotherapy, and chemotherapy.

**Figure 1:**
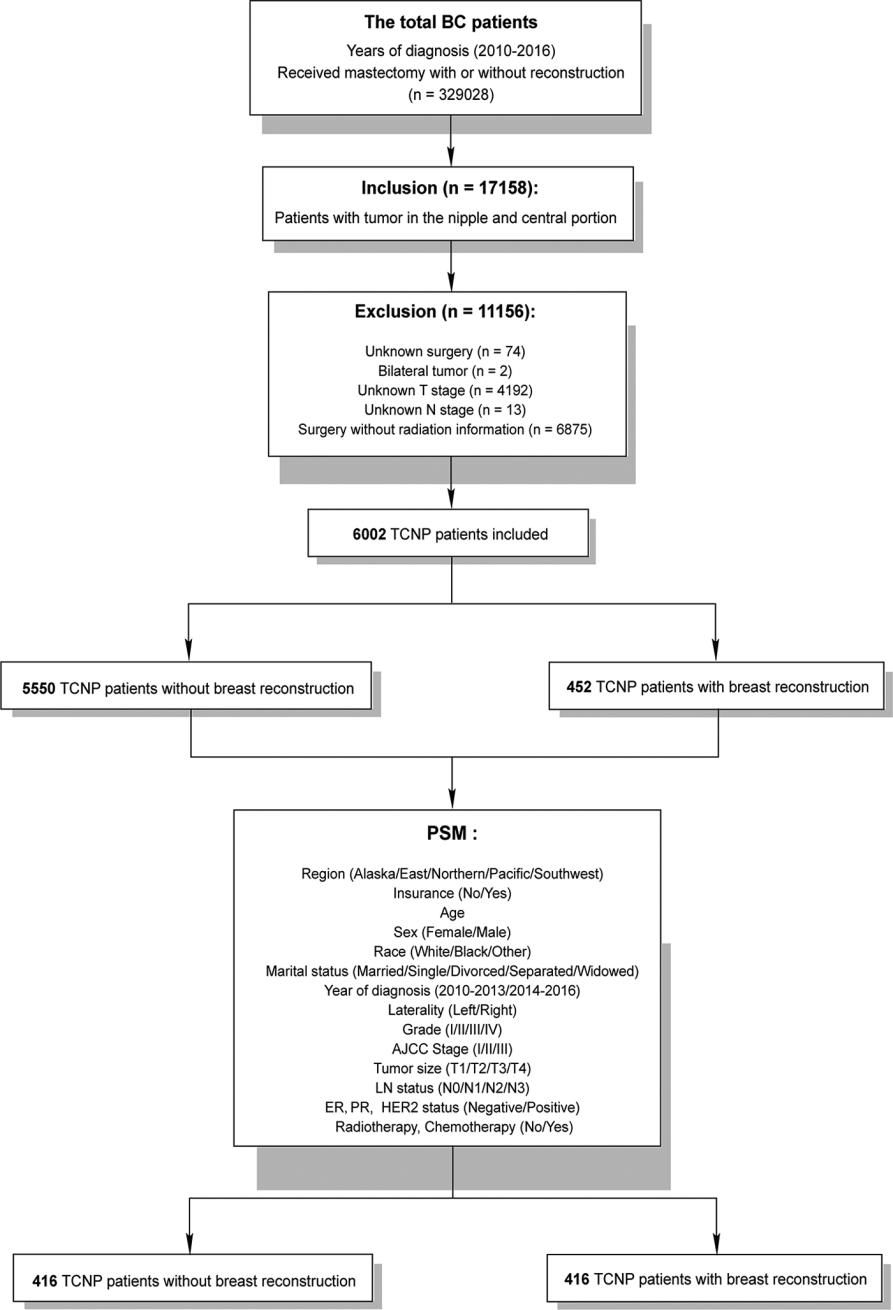
Flow diagram of inclusion criteria and exclusion criteria for analyzing reconstruction influences on tumors in the central and nipple portion patients.

We identified potentially eligible patients based on the following inclusion criteria: year of diagnosis between the years 2010 and 2016, BC as the first malignant cancer diagnosis, and TCNP, having received a mastectomy with or without reconstruction. Reconstruction types included implants, tissues, and combinations of implants and tissues, regardless of whether surgery was immediate or delayed. Implants refer to artificial prostheses. Tissues for reconstruction are defined as human tissues such as muscles or skin. Besides, tissue expander placement at the time of original surgery indicates that reconstruction is planned as part of the first course of treatment. The exclusion criteria were as follows: unknown surgery, bilateral tumor, unknown T stage, unknown N stage, and unknown radiation. Patients who died of other causes were censored on the date of death. The final study sample contained 6,002 TCNP patients, 5,550 patients without reconstruction, and 452 patients with reconstruction eligible for analysis based on our inclusion and exclusion criteria (Figure 1). The tumor stage was categorized according to the American Joint Committee on Cancer (AJCC) staging system, 6th edition.

### Statistical Analyses

Statistical analyses were performed using the SPSS version 23.0 software package (IBM SPSS Statistics, Chicago, IL, USA). The relevant clinicopathological characteristics were compared between mastectomy with the non-reconstruction and reconstruction groups using Pearson’s chi-square test. To balance baseline characteristics between the two groups, the propensity score matching (PSM) analysis was conducted with a ratio of 1.0 to construct a matched sample (Figure 1). Significantly different variables between the two groups were used to generate propensity scores. The caliper was set to 0.02 to conduct a strict match. The standardized mean differences of all variables were shown on the love plot. In this study, BCSS was used as the primary study outcome, indicating the survival time between the dates of diagnosis and the date of death due to BC. The OS was defined as the time from the diagnosis date of BC to the date of death from any cause. The survival curves were plotted using the Kaplan–Meier method. The survival differences were assessed by using the log-rank test. The univariate and multivariate analyses of BCSS and OS were used in the Cox proportional hazards regression model. Parameters with a statistical significance in univariate analysis or with clinical significance were included in the multivariate Cox model. A two-side *p*-value <0.05 was considered statistically significant.

## Results

### Demographic and Clinicopathological Characteristics

Between the years 2010 and 2016, a total of 6,002 BC patients with TCNP matched the study criteria. According to non-reconstruction and reconstruction after mastectomy, all patients with TCNP were divided into two groups: non-reconstruction group (*n* = 5,550, 92.4%) and reconstruction group (*n* = 452, 7.6%). Before PSM, the region, age, sex, marital status, grade, AJCC stage, tumor size, ER status, PR status, HER2 status, and chemotherapy had statistical differences (Table 1). The majority of patients in the non-reconstruction group and reconstruction group came from the East (40.2% and 44.7%) or the Pacific (43.4% and 33.8%). The age median of the non-reconstruction group was older than that of the reconstruction group (63.0 vs. 50.0, *p* < 0.01). Compared with the non-reconstruction group, the reconstruction group had a higher rate of female patients (99.3% vs. 95.9%, *p* < 0.01), married patients (65.6% vs. 58.4%, *p* < 0.01), histological grade III and IV (29.5% vs. 28.5% and 0.9% vs. 0.2%, respectively, *p* < 0.01), and AJCC stage II and III (46.0% vs. 36.2% and 50.7% vs. 20.7%, respectively, *p* < 0.01). In addition, the reconstruction group had a greater number of patients with a tumor size of at least 2 cm than the non-reconstruction group (83.6% vs. 43%, *p* < 0.01). The patients with reconstruction had more LN metastasis (84.7% vs. 42.5%, *p* < 0.01).

**Table 1:** Clinicopathological features of the central and nipple breast cancer patients.

Variables	Data before PSM *N* (%)		*p* *	Data after PSM *N* (%)		*p* *
	Non-reconstruction 5,550 (92.4)	Reconstruction 452 (7.6)		Non-reconstruction 416 (50)	Reconstruction 416 (50)	
Region			<0.01			0.08
Alaska	9 (0.2)	3 (0.7)		0	3 (0.7)	
East	2,231 (40.2)	202 (44.7)		181 (43.5)	180 (43.3)	
Northern	646 (11.6)	67 (14.8)		47 (11.3)	64 (15.4)	
Pacific	2,406 (43.4)	153 (33.8)		169 (40.6)	144 (34.6)	
Southwest	258 (4.6)	27 (6.0)		19 (4.6)	25 (6.0)	
Insurance			0.114			0.12
No	92 (1.7)	12 (2.7)		21 (5.1)	12 (2.9)	
Yes	5,402 (98.3)	433 (97.3)		393 (94.9)	399 (97.1)	
Age			<0.01			<0.01
Median (interquartile range)	63 (54–71)	50 (43–58)		55 (45–64)	50.5 (43–59)	
Sex			<0.01			0.08
Female	5,235 (95.9)	449 (99.3)		416 (100.0)	413 (99.3)	
Male	225 (4.1)	3 (0.7)		0 (0)	3 (0.7)	
Race			0.452			0.05
White	4,429 (80.1)	361 (79.9)		322 (77.4)	335 (80.5)	
Black	530 (9.6)	50 (11.1)		64 (15.4)	42 (10.1)	
Others	571 (10.3)	41 (9.1)		30 (7.2)	39 (9.4)	
Marital status			<0.01			<0.01
Married	3,096 (58.4)	284 (65.6)		237 (58.8)	261 (65.4)	
Single	764 (14.4)	88 (20.3)		65 (16.1)	79 (19.8)	
Divorced/Separated/Widowed	1,443 (27.2)	61 (14.1)		101 (25.1)	59 (14.8)	
Year of diagnosis			0.226			0.94
2010–2013	3,538 (63.7)	301 (66.6)		276 (66.3)	277 (66.6)	
2014–2016	2,012 (36.3)	151 (33.4)		140 (33.7)	139 (33.4)	
Laterality			0.251			0.63
Left	2,857 (51.5)	220 (48.7)		208 (50.0)	201 (48.3)	
Right	2,693 (48.5)	232 (51.3)		208 (50.0)	215 (51.7)	
Grade			<0.01			<0.01
I	1,203 (22.6)	40 (21.6)		17 (4.1)	40 (9.6)	
II	2,598 (48.7)	210 (48.7)		104 (25.0)	207 (49.8)	
III	1,521 (28.5)	178 (29.5)		293 (70.4)	167 (40.1)	
IV	12 (0.2)	4 (0.9)		2 (0.5)	2 (0.5)	
AJCC^a^ Stage			<0.01			<0.01
I	2,396 (43.2)	15 (3.3)		128 (30.8)	11 (2.6)	
II	2,007 (36.2)	208 (46.0)		140 (33.7)	196 (47.1)	
III	1,147 (20.7)	229 (50.7)		148 (35.6)	209 (50.2)	
Tumor Size (cm)			<0.01			<0.01
T1 (<2)	3,166 (57.0)	74 (16.4)		146 (35.1)	69 (16.6)	
T2 (2–5)	1,582 (28.5)	225 (49.8)		138 (33.2)	210 (50.5)	
T3 (>5)	433 (7.8)	111 (24.6)		61 (14.7)	104 (25.0)	
T4^b^	369 (6.6)	42 (9.3)		71 (17.1)	33 (7.9)	
LN status			<0.01			<0.01
N0 (negative)	3,189 (57.5)	69 (15.3)		239 (57.5)	58 (13.9)	
N1 (<3)	1,603 (28.9)	237 (52.4)		118 (28.4)	223 (53.6)	
N2 (4–9)	467 (8.4)	94 (20.8)		39 (9.4)	90 (21.6)	
N3 (>9)	291 (5.2)	52 (11.5)		20 (4.8)	45 (10.8)	
ER status			<0.01			<0.01
Negative	669 (12.2)	84 (18.7)		341 (82.0)	72 (17.3)	
Positive	4,829 (87.8)	365 (81.3)		75 (18.0)	344 (82.7)	
PR status			0.060			<0.01
Negative	1,216 (22.2)	117 (26.1)		364 (87.5)	102 (24.5)	
Positive	4,260 (77.8)	332 (73.9)		52 (12.5)	314 (75.5)	
HER2 status						<0.01
Negative	4,569 (85.9)	335 (76.8)	<0.01	416 (100.0)	323 (77.6)	
Positive	750 (14.1)	101 (23.2)		0 (0)	93 (22.4)	
Radiotherapy			0.327			0.05
Yes	5,354 (96.5)	432 (95.6)		8 (1.9)	18 (4.3)	
No	196 (3.5)	20 (4.4)		408 (98.1)	398 (95.7)	
Chemotherapy			<0.01			<0.01
Yes	2,454 (44.2)	405 (89.6)		74 (17.8)	41 (9.9)	
No	3,096 (55.8)	47 (10.4)		342 (82.2)	375 (90.1)	

*PSM, propensity score matching.*

^a^ *AJCC, American Joint Committee on Cancer.*

^b^ *T4, Tumor of any size with direct extension to the chest wall and/or to the skin (ulceration or skin nodules).*

* *p < 0* *.* *05 was considered statistically significant.*

Given the significant differences between the two groups, a PSM was utilized to balance the most demographic and clinical characteristic distribution. After matching, 416 patients in each group were matched (Table 1). Their age, marital status, grade, AJCC stage, tumor size, ER status, PR status, HER2 status, and chemotherapy had statistically significant differences (*p* < 0.01). No other variables showed notable differences between the non-reconstruction and reconstruction groups (*p* > 0.05). The standardized mean differences of all variables after matching were concentrated around 0, which suggested that the variables were balanced and well-matched (Supplementary Figure 1).

### Kaplan–Meier Analysis with Breast Cancer-Specific Survival and Overall Survival

**Figure 2:**
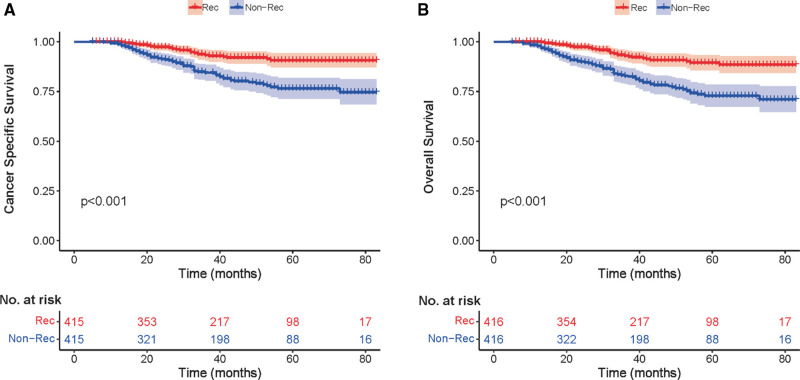
Kaplan–Meier curves comparing breast cancer-specific survival (**A**) and overall survival (**B**) for reconstruction vs. non-reconstruction groups in the exactly matched cohort (*n* = 832) after propensity score matching.

**Figure 3:**
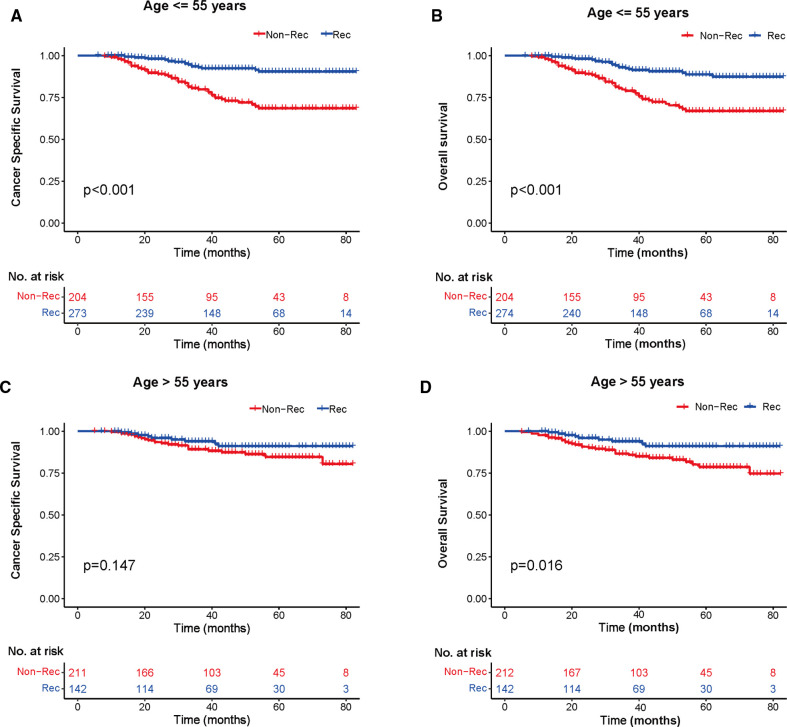
Kaplan–Meier curves of breast cancer-specific survival and overall survival in the reconstruction and non-reconstruction groups stratified by different ages. (**A**) Age ≤55 years, (**B**) Age ≤55 years, (**C**) Age >55 years, and (**D**) Age >55 years.

**Figure 4:**
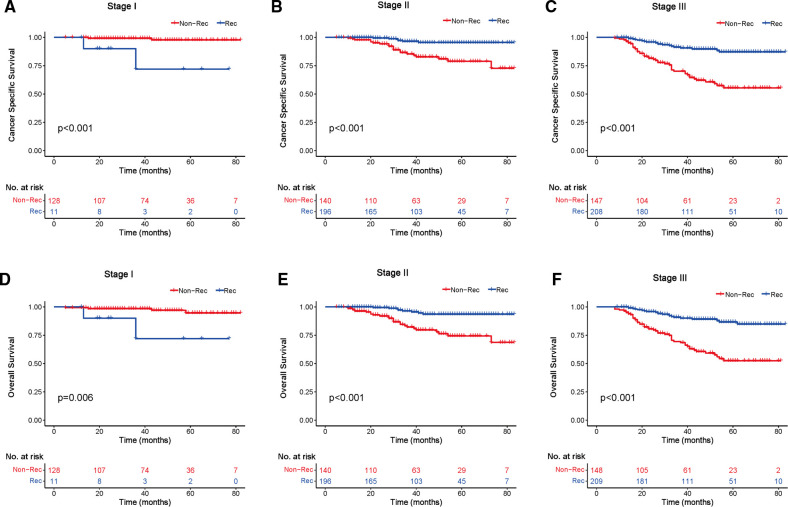
Kaplan–Meier curves of breast cancer-specific survival and overall survival in the reconstruction and non-reconstruction groups stratified by different stages. Breast cancer-specific survival (**A**) stage I, (**B**) stage II, and (**C**) stage III. Overall survival (**D**) stage I, (**E**) stage II, and (**F**) stage III.

**Figure 5:**
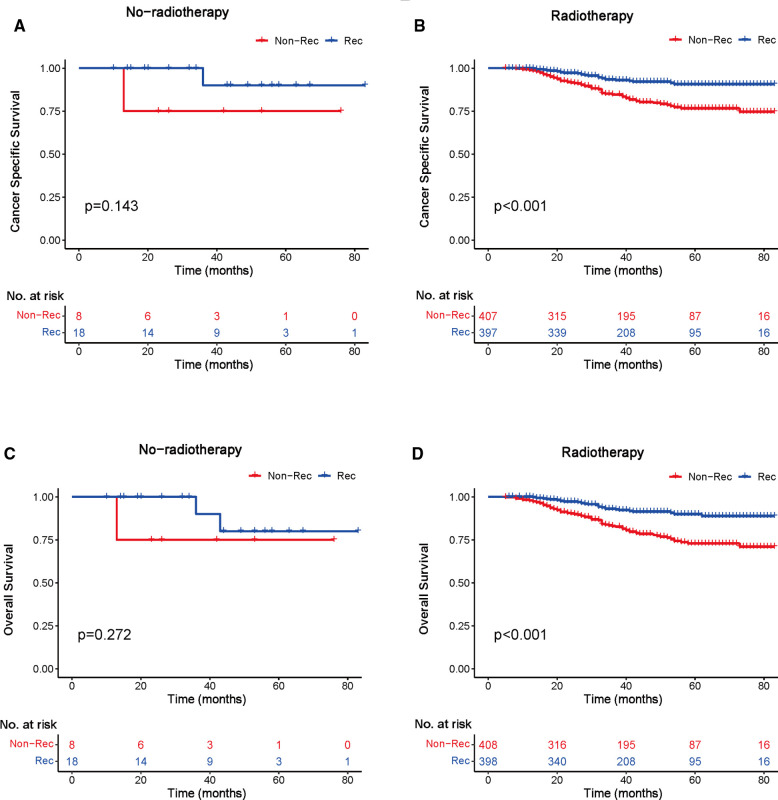
Kaplan–Meier curves of breast cancer-specific survival and overall survival in the reconstruction and non-reconstruction groups with or without radiotherapy. Breast cancer-specific survival (**A**) no radiotherapy and (**B**) radiotherapy; overall survival (**C**) no radiotherapy and (**D**) radiotherapy.

We investigated the outcome of BCSS and OS in patients with TCNP between the non-reconstruction and reconstruction group after mastectomy, using the Kaplan–Meier method. Patients with postmastectomy reconstruction had a significant difference in terms of better BCSS and OS compared with patients with non-reconstruction (*p* < 0.01, Figure 2). We next assessed the prognostic values of the reconstruction group compared with the non-reconstruction group in various subgroups, including age, AJCC stage I to III, and radiotherapy. Compared with the patients without reconstruction therapy, patients with reconstruction had a better prognostic factor for OS in all age groups (*p* < 0.05, Figures 3B,D). However, only reconstruction patients under the age of 55 had improved BCSS (*p* < 0.01, Figure 3A). There was no significant BCSS difference between the reconstruction and non-reconstruction groups of patients older than 55 years (*p* = 0.147, Figure 3C). Patients without reconstruction in stage I showed an improved BCSS and OS compared with patients with reconstruction (*p* < 0.001, Figures 4A,D). Except for AJCC stage I, patients had a better BCSS and OS with postmastectomy reconstruction in stage II–III (*p* < 0.001, Figures 4B,C,E,F). Similar outcomes were present in the radiotherapy of TCNP patients with reconstruction (*p* < 0.01, Figures 5B,D). Patients with TCNP who underwent radiotherapy had improved BCSS and OS from the reconstruction. Also, reconstruction did not affect the BCSS (*p* = 0.143) and OS (*p* = 0.272) of TCNP patients with no radiotherapy (Figures 5A,C).

### Cox Regression Analyses of Prognostic Factors

The Cox proportional hazards model was also used to ascertain the effect of prognostic factors on survival outcomes. The univariable analysis indicated that the reconstruction surgery, AJCC stage III, tumor size T4, LN status N2 and N3, and positive ER, PR, and HER2 status were significantly associated with BCSS and OS (*p* < 0.05, Table 2). However, non-radiotherapy did not affect the disease prognosis. For clinical consideration, some variables were included in the multivariate Cox regression model. The multivariate analysis demonstrated that AJCC stage II–III and positive ER, PR, and HER2 status were maintained as prognostic factors for BCSS and OS (*p* < 0.05, Table 3). Importantly, the reconstruction surgery was not the prognostic factor of TCNP patients (*p* > 0.05, Table 3). The BCSS and OS of patients with or without reconstruction revealed no significant differences. In addition, radiotherapy was associated with better BCSS and OS in patients with TCNP after reconstruction (*p* < 0.05, Table 3).

**Table 2:** Univariable Cox proportional hazard regression model of cancer-specific survival and overall survival.

Variables	CSS		OS	
	HR (95% CI)	*p* *	HR (95% CI)	*p* *
Reconstruction				
No	Ref		Ref	
Yes	2.76 (1.76–4.30)	<0.01	2.78 (1.83–4.21)	<0.01
Region				
Alaska	Ref		Ref	
East	NA	NA	NA	NA
Northern	5.45 (0.75–39.50)	0.09	3.02 (0.74–12.43)	0.13
Pacific	4.40 (0.57–33.80)	0.16	2.57 (0.58–11.30)	0.21
Southwest	5.20 (0.71–37.89)	0.10	3.09 (0.75–12.73)	0.12
Insurance				
No	Ref		Ref	
Yes	0.67 (0.27–1.64)	0.38	0.79 (0.32–1.93)	0.60
Age	0.99 (0.97–1.01)	0.17	1.00 (0.98–1.01)	0.76
Sex				
Female	Ref		Ref	
Male	NA	NA	NA	NA
Race				
White	Ref		Ref	
Black	2.49 (1.57–3.95)	<0.01	2.20 (1.41–3.42)	<0.01
Others	0.71 (0.29–1.77)	0.47	0.84 (0.39–1.82)	0.84
Marital status				
Married	Ref		Ref	
Single	1.02 (0.59–1.75)	0.95	0.99 (0.60–1.64)	0.98
Divorced /Separated /Widowed	1.20 (0.62–2.32)	0.60	1.20 (0.65–2.21)	0.56
Year of diagnosis				
2010–2013	Ref		Ref	
2014–2016	0.82 (0.44–1.56)	0.55	0.70 (0.37–1.31)	0.26
Laterality				
Left	Ref		Ref	
Right	0.91 (0.98–1.46)	0.91	1.04 (0.71–1.51)	0.85
Grade				
I	Ref		Ref	
II	NA	NA	NA	NA
III	NA	NA	NA	NA
IV	NA	NA	NA	NA
AJCC^a^ Stage				
I	Ref		Ref	
II	2.87 (1.00–8.22)	0.05	2.44 (1.02–5.82)	0.45
III	6.91 (2.52–18.97)	<0.01	5.03 (2.19–11.58)	<0.01
Tumor Size (cm)				
T1 (<2)	Ref		Ref	
T2 (2–5)	1.59 (0.85–2.96)	0.15	1.54 (0.87–2.72)	0.14
T3 (>5)	2.28 (1.17–4.43)	0.15	2.18 (1.19–4.00)	0.12
T4^b^	4.22 (2.18–8.15)	<0.01	3.77 (2.05–6.95)	<0.01
LN status				
N0 (negative)	Ref		Ref	
N1 (<3)	1.54 (0.90–2.65)	0.12	1.27 (0.79–2.06)	0.32
N2 (4–9)	2.30 (1.25–4.24)	<0.01	1.84 (1.06–3.20)	0.03
N3 (>9)	4.35 (2.34–8.09)	<0.01	3.17 (1.78–5.66)	<0.01
ER status				
Negative	Ref		Ref	
Positive	0.29 (0.18–0.47)	<0.01	0.28 (0.18–0.44)	<0.01
PR status				
Negative	Ref		Ref	
Positive	0.25 (0.15–0.43)	<0.01	0.23 (0.14–0.38)	<0.01
HER2 status				
Negative	Ref		Ref	
Positive	0.23 (0.07–0.73)	<0.01	0.27 (0.10–0.73)	0.01
Radiotherapy				
Yes	Ref		Ref	
No	0.91 (0.29–2.87)	0.87	0.78 (0.29–2.13)	0.63
Chemotherapy				
Yes	Ref		Ref	
No	5.07 (1.61–16.01)	<0.01	2.43 (1.13–5.23)	0.23

*CSS, cancer-specific survival; OS, overall survival; HR, hazard ratio; CI, confidence interval.*

^a^ *AJCC, American Joint Committee on Cancer.*

^b^ *T4, Tumor of any size with direct extension to the chest wall and/or to the skin (ulceration or skin nodules).*

* *p < 0* *.* *05 was considered statistically significant.*

**Table 3:** Multivariable Cox proportional hazard regression model of cancer-specific survival and overall survival.

Variables	CSS		OS	
	HR (95% CI)	*p* *	HR (95% CI)	*p* *
Reconstruction				
No	Ref		Ref	
Yes	0.76 (0.41–1.39)	0.37	0.77 (0.43–1.36)	0.36
Age	0.99 (0.97–1.01)	0.23	1.00 (0.98–1.01)	0.55
Race				
White	Ref		Ref	
Black	1.60 (0.98–2.62)	0.06	1.44 (0.90–2.32)	0.13
Others	0.62 (0.25–1.55)	0.30	0.72 (0.33–1.58)	0.41
Marital status				
Married	Ref		Ref	
Single	0.89 (0.51–1.54)	0.67	0.96 (0.58–1.61)	0.89
Divorced /Separated /Widowed	0.72 (0.41–1.25)	0.24	0.71 (0.42–1.19)	0.19
AJCC^a^ Stage				
I	Ref		Ref	
II	4.11 (1.35–12.52)	0.01	5.57 (2.05–15.18)	<0.01
III	14.05 (4.71–41.89)	<0.01	16.90 (6.18–46.21)	<0.01
ER status				
Negative	Ref		Ref	
Positive	0.36 (0.17–0.74)	<0.01	0.40 (0.20–0.80)	<0.01
PR status				
Negative	Ref		Ref	
Positive	0.40 (0.18–0.86)	0.02	0.33 (0.16–0.69)	<0.01
HER2 status				
Negative	Ref		Ref	
Positive	0.23 (0.07–0.79)	0.02	0.28 (0.09–0.83)	0.02
Radiotherapy				
No	Ref		Ref	
Yes	0.28 (0.08–0.91)	0.04	0.27 (0.09–0.75)	0.01
Chemotherapy				
No	Ref		Ref	
Yes	1.70 (0.48–6.00)	0.41	0.85 (0.35–2.07)	0.72

*CSS, cancer-specific survival; OS, overall survival; HR, hazard ratio; CI, confidence interval.*

^a^ *AJCC, American Joint Committee on Cancer.*

* *p < 0* *.* *05 was considered statistically significant.*

## Discussion

This retrospective analysis of 6,002 cases in the SEER database is a novel research to decipher the role of postmastectomy reconstruction in the survival of BC patients with TCNP. Compared with patients without reconstruction, TCNP patients with reconstruction exhibited no significant influence in BCSS and OS after PSM, especially when younger (age ≤55), with AJCC stage II–III and with radiotherapy. Importantly, the multivariate analysis revealed that reconstruction did not affect the prognosis and survival of TCNP patients. The effect on survival observed in the TCNP patients with reconstruction highlights the safety and applicability of reconstruction in the management of postmastectomy treatment.

Breast reconstruction is the main step in the management of postmastectomy treatment, which is beneficial to psychological health and alleviates pain (13). Postmastectomy reconstruction can be divided into two, namely, immediate surgery and delayed surgery, most of which use autologous fat tissue or breast implants to shape breast appearance (14). In recent years, the overall rates of breast reconstruction have increased (15). However, ensuring tumor safety and minimizing the frequency of complications based on the cosmetic results of breast reconstruction remain key goals of multidisciplinary collaboration among physicians (16). Recent studies have confirmed the irrelevance between breast reconstruction and tumor recurrence and prognosis. Platt et al. completed a 20-year follow-up in a large cohort with invasive BC and found that women undergoing reconstruction had improved survival benefits compared with women with only mastectomy (17). Within 5 years of invasive BC diagnosis, breast reconstruction reduced the risk of death by at least 17% (17). Based on the SEER database, Bezuhly et al. found that immediate breast reconstruction was associated with decreased BC-specific mortality, particularly among younger women (18). Using the data from The Johns Hopkins Hospital comprehensive cancer registry, Siotos et al. concluded that BC patients with reconstruction had better OS and a similar recurrence rate with mastectomy-only patients (19). All these studies proved the survival benefit among patients with breast reconstruction, which alleviated long-term oncologic safety concerns on breast reconstruction.

The primary tumor location also plays a key role in BC prognosis (6). The biological behavior and survival of breast tumors in different primary sites are different. Rummel et al. analyzed data from the Clinical Breast Care Project and demonstrated that tumors in the central quadrant had significantly higher tumor stage and size, metastatic lymph nodes, and mortality (20). They attributed this to the difficulty of mammography for central tumors, resulting in larger tumor size at diagnosis and a less favorable prognosis. However, Siotos et al. found that tumors in the central portion had the highest mortality (16.9%) and no low recurrence (4.3%), and positive lymph nodes were present in 33.9% nipple locations and 26.9% central locations, compared with other peripheral locations in the Johns Hopkins Hospital database (6). The central and nipple tumor sites might be associated with survival and be an important characteristic affecting the prognosis of patients with breast cancer. Using the National Cancer database, Desai et al. concluded that patients with invasive breast cancer located in the nipple, central breast, and axillary tail had the highest risk of positive axillary lymph nodes, independent of patient age, tumor grade, biologic subtype, histology, and size (21). Consistent with these findings, Zhang et al. proved that central tumors were also significantly associated with axillary lymph node metastasis (22). Particularly, Yang et al. concluded that tumors located in the medial, central, and lower quadrants showed adverse survival (23). Lower and central breast tumors were more able to harbor disease in the internal mammary chain (24). In addition, Ji et al. revealed that TCNP was associated with older age, larger tumor size, TNM stage II–III, and lymph node metastasis, which had inferior outcomes on BCSS and OS, compared with tumors in the breast peripheral quadrant (7). This research also showed that patients with TCNP also underwent more mastectomies. Thus, emerging women who have mastectomies are opting for breast reconstruction after their tumors are in remission. However, opinion on whether breast reconstruction is suitable for TCNP patients remains few in number. In this study, we investigated whether breast reconstruction would affect the prognosis of BC patients with TCNP. With the Kaplan–Meier method, patients undergoing postmastectomy reconstruction had no significantly worse survival in BCSS and OS, compared with patients with non-reconstruction. Particularly with younger age, AJCC stage II–III, and radiotherapy, TCNP patients undergoing reconstruction showed great survival advantage. More importantly, some variables containing common clinical factors were evaluated in a multivariate Cox regression model. The AJCC stage II–III, positive ER, PR, HER2 status, and radiotherapy were maintained as prognostic factors for BCSS and OS. Remarkably, patients with reconstruction showed no statistical differences in BCSS and OS compared with patients without reconstruction. Thus, reconstruction surgery was not a risk factor for TCNP patients.

Age is generally acknowledged to be a risk factor related to the prognosis for undergoing breast reconstruction (25). Older age, Asian race, bilateral mastectomy, and higher stage of the disease also proved to be independent risk factors for not receiving immediate breast reconstruction (26). Fewer older patients received breast reconstruction, probably due to patient concerns about tumors and physical condition, and the breast surgeon perception of the disease stage (27–29). However, our study demonstrated that breast reconstruction did not affect the prognosis of TCNP patients with higher stages. This might establish a more detailed foundation for patients and surgeons to understand breast reconstruction in disease survival. In addition, postmastectomy radiotherapy was found to decrease local recurrence and improve the survival of patients with node-positive disease (30). But radiotherapy has also been found to increase the risk of complications and negatively affect reconstructive and cosmetic outcomes of the breast (31). Meanwhile, the reconstructed breast could also increase the complexity of radiotherapy delivery. Thus, the postmastectomy reconstruction of patients at radiotherapy risk was a difficult clinical decision (16). However, in the setting of postmastectomy radiation therapy, the frequency of immediate reconstruction was increasing (32). Despite the increasing receipt of postmastectomy radiotherapy, the strongly recommended radiotherapy cohort maintained a consistent increase in breast reconstruction (33). Our results constituted a broad and diverse set of influencing factors in the SEER database and provided more convincing evidence that TCNP patients with radiotherapy might not be a relative contraindication for clinical decision-making on postmastectomy reconstruction.

This present study also has some limitations. First, this retrospective study used PSM analysis, showing a relatively small sample size. The fitting degrees of the propensity score estimation model might have influenced the outcomes. It is necessary to use larger sample sizes for further independent validation and prognostic analysis. Subsequent evidence-based medicine and more prospective studies such as RCTs are needed to confirm the actual effects of breast reconstruction on TCNP patient survival. Second, the SEER database does not cover all variables such as neoadjuvant chemotherapy information, comorbidities, and complications, which interfered with the conduct of an in-depth analysis in the utilization of breast reconstruction (12). According to the breast surgery codes, reconstruction is mainly divided into three categories: tissue, implant, and combined (tissue and implant). More classification details on reconstruction, such as immediate and early delayed reconstruction, are needed. In addition, the subjective advice of the surgeon on reconstruction, patient preferences, and financial situation could influence the final decision, which was the unknowns using the SEER database. Lastly, the United States-based SEER database might not be applicable all over the world. More importantly, external validation from other populations is still needed to eliminate unwanted selection bias.

## Conclusion

To sum up, this study demonstrated that there were no survival differences between TCNP patients undergoing postmastectomy reconstruction and TCNP patients with non-reconstruction. This survival advantage of TCNP patients receiving postmastectomy reconstruction persisted especially in age ≤55 years, AJCC stage II–III, and during radiotherapy. Thus, postmastectomy reconstruction could benefit patients with TCNP to enhance body image, self-esteem, and life quality without compromising oncologic and survival outcomes.

## Data Availability

The original contributions presented in the study are included in the article/Supplementary Material; further inquiries can be directed to the corresponding author/s.
