# Supplementary figures and images for: Breast Reconstruction Does Not Affect the Survival of Patients with Breast Cancer Located in the Central and Nipple Portion: A Surveillance, Epidemiology, and End Results Database Analysis
Source: Front Surg. 2022 May 17;9:855999. doi: 10.3389/fsurg.2022.855999 (PMC9406515; doi:10.3389/fsurg.2022.855999)

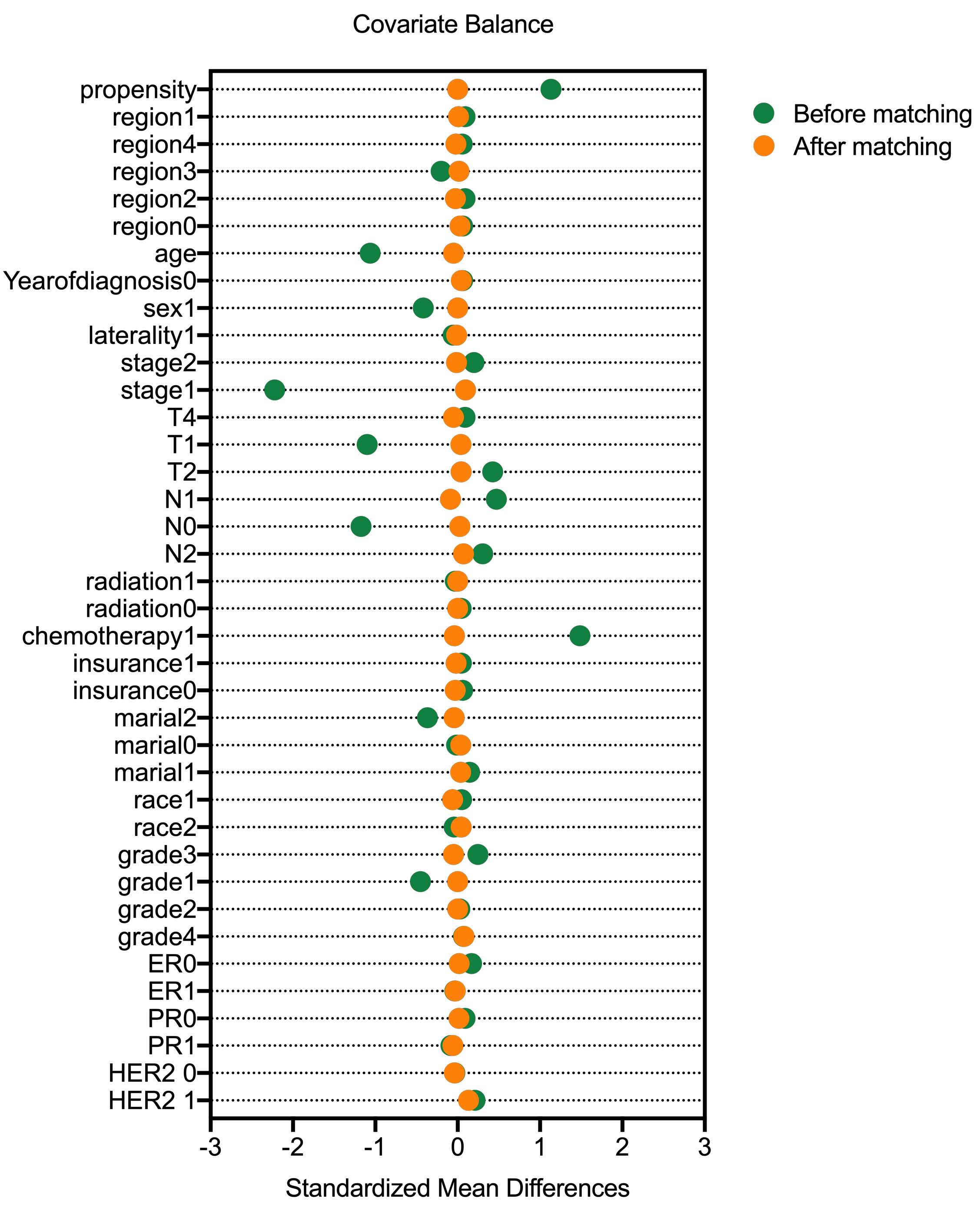

Supplement: Supplementary file 1 [file Image_1_v1.tiff]
